# Age-Dependent Performance on Pro-point and Anti-point Tasks

**DOI:** 10.3389/fpsyg.2018.02519

**Published:** 2018-12-17

**Authors:** Elijah K. Li, Shannon Lee, Saumil S. Patel, Anne B. Sereno

**Affiliations:** ^1^Department of Neurobiology and Anatomy, McGovern Medical School, The University of Texas Health Science Center at Houston, Houston, TX, United States; ^2^Department of Psychological Sciences, Rice University, Houston, TX, United States; ^3^School of Behavioral and Brain Sciences, University of Texas at Dallas, Dallas, TX, United States; ^4^Department of Neuroscience, Baylor College of Medicine, Houston, TX, United States; ^5^Department of Psychological Sciences, Purdue University, West Lafayette, IN, United States; ^6^Weldon School of Biomedical Engineering, Purdue University, West Lafayette, IN, United States

**Keywords:** cognitive development, saccades, cognitive control, sensorimotor control, prefrontal cortex

## Abstract

Changes in prefrontal cortex are thought to be responsible for many of the characteristic behavioral changes that are seen during adolescence and late adulthood. Disruption of prefrontal cortex is an early sign for many developmental, neurological, and psychiatric disorders. Goal directed eye movements, such as Anti-saccades, have been shown to have high sensitivity as a gross assessment of prefrontal lobe function. Previous studies on the developmental changes of saccades across age have shown that stimulus-driven and goal-directed eye movements follow a *U*-shaped trend with peaks in performance occuring during adolescence. Using novel tablet-based pointing tasks, modeled on eye movement tests, this study aims to provide a preliminary understanding of how age affects manual pointing performance, in order to more easily track behavioral changes of the prefrontal cortex. In this study, 82 participants between the ages of 10 and 63 were recruited to participate. Results show that similarly to saccades, manual pointing responses are age dependent with fastest response times found during late adolescence to early adulthood (*U*-shaped curves). Importantly, we also demonstrated significant differences in the effect of age in stimulus-driven (Pro-point) and goal-directed (Anti-point) pointing tasks. The effect of age on response time (RT) is greater on Anti-point compared to Pro-point task (with a 79 ms greater mean decrease during early development and a 148 ms greater mean increase during later aging). Further, for Pro-point task, the *U*-shaped curve flattens at about 45 years whereas for Anti-point task the *U*-shaped curve continues up to the maximum age tested (about 60 years). This dissociation between age-related changes in sensorimotor and cognitive performance suggests independent development of associated brain circuity. Thus, changes of performance in disease that are specific for age and task may be able to help identify brain circuitry involved. Finally, given that these tablet-based pointing tasks show similar age-related patterns reported previously with eye-tracking technology, our findings suggest that such tablet-based tasks may provide an inexpensive, quick, and more practical way of detecting neurological deficits or tracking cognitive changes.

## Introduction

Prefrontal cortex (PFC) is thought to be essential to various executive functions critical for goal directed behavior, including cognitive control, planning, memory, and attention (Moriguchi and Hiraki, 2013; Yuan and Raz, 2014; Ouhaz et al., 2018). PFC is greatly expanded in primate (Fuster, 1988) and its dysfunction is associated with many human neurological, psychiatric, and developmental disorders (Amaral et al., 2008; Goto et al., 2010; Hoban et al., 2016). PFC is one of the last regions of the brain to mature (Giedd et al., 1999; Sowell et al., 2001; Arain et al., 2013), with development not completed until nearly age 25 (Casey et al., 2008; Arain et al., 2013). The relatively late developmental changes in PFC have been suggested to be one explanation for the behavioral immaturity and risk-taking behavior in adolescence (Arain et al., 2013). Changes in PFC in adolescence are thought to be critical for the adolescent’s increased ability to demonstrate impulse control, emotional regulation, and in general, better executive functioning (Zilbovicius et al., 1995; Yurgelun-Todd, 2007; Caballero et al., 2016). In addition, age-related deterioration and dysfunction of PFC is thought to be responsible for the onset and progression of executive dysfunction in adulthood (Arnsten and Goldman-Rakic, 1985; Salat et al., 2001).

Much research has focused on the role of PFC in regulating eye-movement related activity (Kojima, 1980; Boch and Goldberg, 1989; Funahashi, 2014). Studies have also shown that saccade dynamics vary as a function of age (Munoz et al., 1998). When Pro-saccade and Anti-saccade performance are assessed as a function of age, robust asymmetric *U*-shape functions are produced for both tasks. This is likely due to the natural processes of development during early adolescence and more gradual degeneration during adulthood. In addition to being an indicator of sensorimotor performance, saccade movement has also been shown to be an effective way of assessing cognitive abilities (Liversedge and Findlay, 2000; Bowling and Draper, 2014; Paolozza et al., 2015; Zagermann et al., 2016; Shaikh and Zee, 2017). In particular, an Anti-saccade requires voluntary inhibition of a natural reflex which demands proper functioning of the frontal lobe and other brain structures associated with higher order executive functioning (Sereno and Bolding, 2017).

Connolly et al. (2000) found that pointing tasks activate a similar frontoparietal network as recruited for saccades, with the recruitment of some additional frontoparietal areas for Anti-pointing tasks. Similar to the literature regarding saccade dynamics, pointing tasks seem to be influenced by age as well. Hertzum and Hornbæk (2010) show that between young (12–14), adult (25–33), and elderly (61–69) subjects, the adult subjects perform pointing tasks quicker and with fewer errors than the other two groups. Additionally, a cross-sectional study done by Teeken et al. (1996) revealed a significant effect of age on pointing tasks as subjects moved significantly slower with greater age. Thus, previous research concerning age related saccade dynamics and pointing tasks implies that the development of goal-directed cognitive abilities (those that are believed to be involved in Anti-saccade and Anti-pointing tasks) are related to age. However, no study exists monitoring age-related changes in stimulus-driven and goal-directed pointing responses from adolescence to late adulthood.

The first goal of this study is to compare age-related changes in stimulus-driven and goal-directed functioning on pointing tasks and to establish a preliminary age-based standard for these tasks. The pattern of associations and dissociations in stimulus-driven and goal-directed performance across age provides insight into the brain circuity underlying these behaviors and can help identify specific changes that might occur in disease. Performance on various cognitive tasks is often used as a marker for normal neurophysiological development (Grant et al., 2001; Baillargeon et al., 2012). Visual and cognitive functions first improve or develop during adolescence (Luna et al., 2004) and then slow down with age as control over reflexes decline (Ridderinkhof and Wijnen, 2011; Wild-Wall et al., 2011). Older adults are expected to have slower saccade reaction times because of the multiple neural systems that are involved during a saccade, all of which are at risk for age-related declines (Pratt et al., 1997; Butler and Zacks, 2006; Eckert, 2011). While motor skills performance ultimately deteriorates with age, it has been found that the ability to correctly inhibit motor control, or to perform voluntary goal-directed movements is relatively unaffected. However, this ability does slow down with age more than stimulus-driven responses (Rossit and Harvey, 2008; Verneau et al., 2015).

The second goal of this study aims to relate pointing responses to previous eye movement findings, in part to test new tablet-based pointing tasks as a possible replacement tool. Terao et al. (2017) show that there is a close spatial and temporal coupling between the eyes and hand movements. This study uses novel tablet-based pointing tasks, modeled after eye tracking technology, to measure stimulus-driven and goal-directed abilities as a function of age (Zhang et al., 2013; Sereno et al., 2017, 2018). Establishing a preliminary normative standard for these tablet-based tasks, especially in adolescence, a stage with rapid brain changes, will help discriminate between what is normal and what is abnormal development. If these tablet-based pointing tasks result in an age-related pattern of pointing performance similar to saccadic eye-tracking technology, they could provide an inexpensive, quick, and more practical way of detecting and tracking stimulus-driven and goal-directed changes or neurological deficits in these behaviors.

In sum, because of close coupling between eye and hand movements and the known slowing of motor skills as one ages (Goggin and Meeuwsen, 1992; Pratt et al., 1994), we hypothesized that there should be parabolic *U*-shaped curves, similar to what Munoz et al. (1998) found for Pro-saccade and Anti-saccade tasks and what Hertzum and Hornbæk (2010) found for pointing performance, suggesting a rapid development of stimulus-driven and goal-directed functioning during adolescence and a gradual decline as a person gets older. In addition, we hypothesized that there would be significant differences in stimulus-driven and goal-directed functioning, with goal-directed processes more impacted than stimulus-driven in older adults, measured as response time changes on the Pro-point and Anti-point task, respectively.

## Materials and Methods

### Participants

All participants in this study were recruited by word of mouth and flyers and gave informed consent or assent with parental consent and the study was approved by the University of Texas at Houston Committee for the Protection of Human Subjects in accordance with the Declaration of Helsinki. Participants with current injury or history of head trauma in the past 2 years were excluded. There were a total of 82 participants (36 male and 46 female) between the ages of 10–63. The demographics of each group are listed in Table 1. Although some previous studies examining age-related changes in arm movements have used only adults with as few as two divisions (Sarlegna, 2006; Verneau et al., 2015), we sought to examine age across a broader age range and with a more fine-grained analysis. Hence, the sample was divided by age using decades with additional divisions in the youngest group in order to examine the known rapid changes during early adolescence (Arain et al., 2013). These age divisions were modeled after an earlier study done by Munoz et al. (1998) that revealed rapid developmental changes during adolescence.

**Table 1:** Demographic Characteristics.

Age range	Mean age (± SD)	Number of Subjects	Male	Female
10–12	10.6 ± 0.8	7	3	4
13–14	13.4 ± 0.5	5	2	3
15–17	16.0 ± 1.3	9	3	6
18–22	20.1 ± 1.7	12	3	9
23–29	26.2 ± 2.5	7	5	2
30–39	34.1 ± 2.8	12	6	6
40–49	45.6 ± 3.1	15	7	8
50–59	54.6 ± 3.5	12	6	6
60+	61.3 ± 1.5	3	1	2
Total		82	36	46

### Apparatus

Experiments were performed using two tablet-based pointing tasks, Pro-point and Anti-point, modeled after eye tracking tasks that have been shown to be sensitive in measuring subtle stimulus-driven and goal-directed deficits (Fischer et al., 2016; Williams et al., 2017). The tasks were performed on an iPad 2 (model MC769LL; iOS 6.1.3) with screen dimensions of 9.50 in by 7.31 in and refresh of 60 Hz.

### Testing Procedure

The subjects were tested sitting down at a table in a quiet, enclosed room. The iPad was placed on the table and the test administrator instructed the subjects on how to perform each task before each block of trials. Furthermore, the order of the two tasks was counterbalanced within age divisions so that half the subjects performed the Pro-point test first and the other half performed the Anti-point test first.

#### Pro-point and Anti-point Tasks

The Pro-point and Anti-point tasks were administered in two separate blocks until 48 correct trials were completed in each task with a maximum of 60 trials, consistent with previous studies using this novel tablet-based pointing task (Fischer et al., 2016). On each trial, the iPad first displayed a fixation circle with a diameter of 1.4 cm (about 2.4°) surrounded (left, right, up, and down) by four square boxes with side lengths of 0.8 cm (about 1.4°) and centers positioned 4.0 cm (about 7.0°) away from the fixation circle. To initiate the trial, the participant had to hold the fixation circle with the index finger of their dominant hand for 480 ms until a white square stimulus appeared at one of the four boxes. The participant then had to lift their finger from the fixation circle and tap the correct goal box as quickly and as accurately as possible. On the Pro-point task the correct goal location was the box with the white square stimulus, on the Anti-point task, it was the box opposite the one with the white square stimulus. Trials containing errors were repeated. An error included any touch response that had a distance that was greater than 1.9 cm away from the correct goal location.

The Pro-point task, paralleling a Pro-saccade, measures stimulus-driven function as the subject is required to make a reflexive movement (McDowell et al., 2008). In each trial, the subject must execute a reflexive, sensory driven response by pointing to the white square. The Anti-point task, paralleling an Anti-saccade, measures goal-directed function as the subject is required to inhibit a reflexive response to the sensory stimulus and generate a voluntary, internally generated, intentional response, utilizing the frontal lobe in the process (Nieuwenhuis et al., 2000).

Although errors are a central dependent measurement in Anti-saccade tasks, measurement of response time (RT, in ms) is better suited for analysis in a pointing task like the one used in this study because these movements are not ballistic like those of a saccade and so subjects make very few, if any, errors (less than 1% errors in the Anti-point). The key RT dependent variable was defined as the duration of time between when the stimulus appears and when the subject makes a touch response.

### Statistical Analyses

All participants except one subject completed 48 correct trials. When an error was made, the pointing application marked the trial as an error and re-presented any error trials in a random order at the end of the task, up to a maximum of 60 trials. There was only one subject that completed 60 trials while only completing 43 correct trials. Removing this subject’s data did not significantly alter any of the findings reported here.

When analyzing the RT data for the Pro-point and Anti-point tasks, any trial containing an error was removed from analysis. This removed 0.57 and 0.93% of trials for Pro-point and Anti-point tasks, respectively. Additionally, consistent with the study done by Fischer et al. (2016), RT for any trials that were greater than 2.75 standard deviations from the mean were trimmed iteratively until all remaining trials were within 2.75 standard deviations of the mean. An additional 3.51 and 3.86% of trials were removed from the Pro-point and Anti-point tasks, respectively.

To evaluate the effect of gender, task, and age, a mixed model analysis was performed in SPSS 18 (IBM) with Task and Gender as the fixed nominal factors and Age as a covariate fixed factor. In separate models, we used individual subject’s mean RT of filtered data (mean model) as well as median RT of unfiltered data (median model) as the dependent variable. Task was also defined as a repeated measures variable. Starting from a full factorial model, models were iteratively pruned to remove nonsignificant terms. To compare the magnitude of early developmental changes (decreases leading up to peak performance) and aging changes (increases following peak performance) across tasks, two additional variables were calculated for each task: (1) Early developmental decrease: The maximum decrease in mean RT between any two ages (DRT) and (2) Aging increase: The maximum increase in mean RT between any two ages (IRT). A *t*-test using pooled variance was conducted to examine the mean difference between Pro-point and Anti-point tasks for each of the above variables (Altman, 1991). The 95% confidence interval is reported along with the mean difference in DRT and IRT across the two tasks.

**FIGURE 1:**
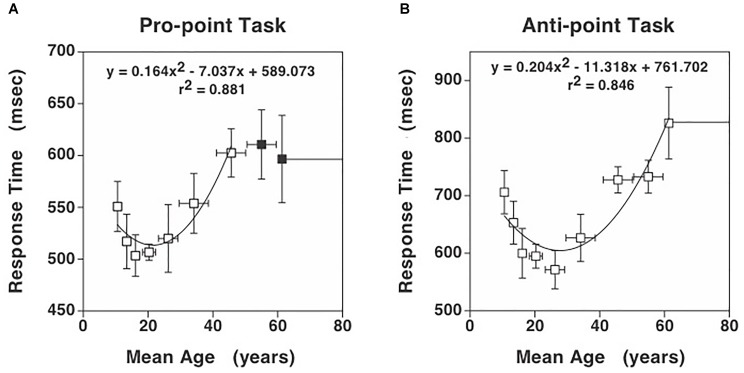
Response times for the Pro-point and Anti-point tasks. **(A)** Response times in Pro-point task as a function of the center of the age range shown in Table 1. Also shown is the quadratic curve fit to the data. **(B)** Response times in Anti-point task as a function of the center of the age range shown in Table 1. Closed symbols indicate data beyond which the partial *F* analysis indicated that the contribution of the quadratic regressor was not significant (see Figure 2). Only the data represented by open symbols were used for the curve fit. The vertical error bars represent ± 1 SD and the horizontal bars represent the age range around the center. Note that the vertical axis has different scales in **(A)** and **(B)** to clearly illustrate the quadratic aspect of the data. Additionally, at these scales, the SD in **(A)** and **(B)** are comparable.

**FIGURE 2:**
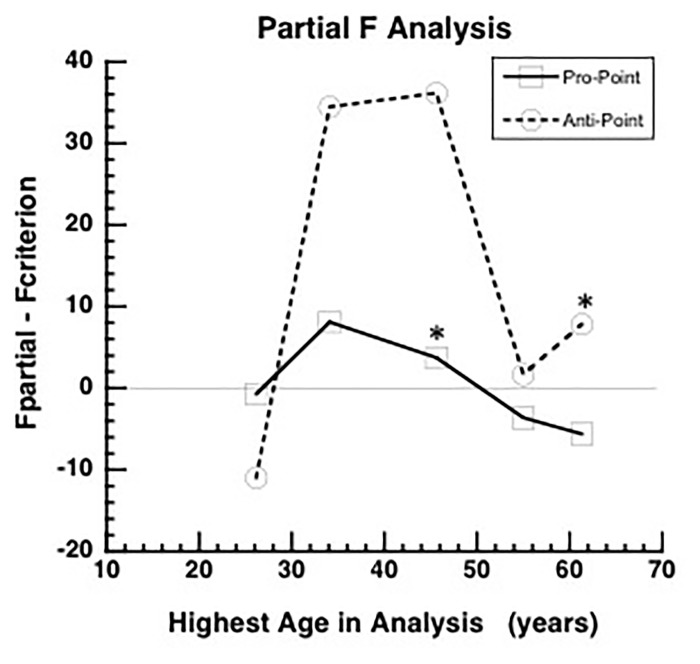
Partial *F* analysis to determine the highest age, indicated by an asterisk, for which the quadratic term is significant in the full model (equation 1). The quadratic term is significant if the difference between F_partial_ and $F_{0.05}^{\left(\text{p}-\text{g},\text{n}-\text{k}\right)}$ (*F*_criterion_) is greater than zero. The difference between F_partial_ and *F*_criterion_ increases initially as data from higher ages are added to the analysis and then after reaching a peak, the difference decreases and becomes negative for Pro-point task around mean age of 45 years (asterisk). The difference remains positive up to the highest age tested (asterisk) for the Anti-point task. Therefore, the fitted curve shown in Figure 1A only utilized data up to mean age of 45 years and the fitted curve shown in Figure 1B utilized all the data.

Linear regression was used to model the effect of age on Pro-point and Anti-point RT. Pro-point and Anti-point responses were analyzed separately. The full regression model was:

(1) $$r=b0+b1x+b2x^{2}$$

where, *b*’s are the regression coefficients, *r* is the response time, and *x* is age. Prior to regression analyses, mean age was removed from the age variable (*x*) to reduce the effect of collinearity between linear and quadratic regressors on regression results. Examination of our data suggested that RT in the Pro-point task may not be related quadratically to age beyond about 45 years of age (see Figure 1A). To evaluate the contribution of the quadratic regressor, we used a reduced model as below:

(2) r=b0+b1x

and calculated a partial *F* statistic as below:

(3) fpartial=SSEfull−SSEreduced(p−g)SSEfull(n−k)

where, *p* is the number of coefficients in the full model minus 1 ( = 2), *g* is the number of coefficients in reduced model minus 1 ( = 1), *n* is the number of observations, and *k* is the number of coefficients in full model ( = 3). If F_partial_ > $F_{0.05}^{\left(\text{p}-\text{g},\text{n}-\text{k}\right)}$ then the contribution of the quadratic regressor can be considered significant at 0.05 level. We computed partial *F* statistic as a function of the highest age used in the analysis data set and plotted the difference between F_partial_ and $F_{0.05}^{\left(\text{p}-\text{g},\text{n}-\text{k}\right)}$ in Figure 2.

**FIGURE 3:**
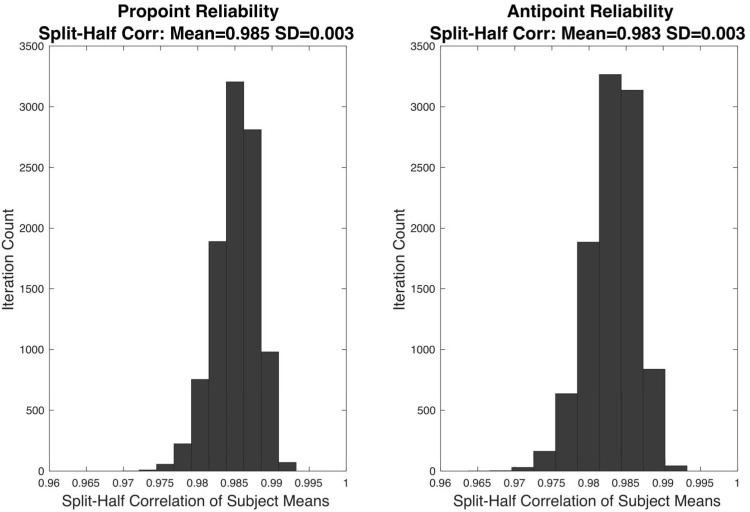
The results of split-half reliability test are shown for Pro-point and Anti-point tasks. Each panel shows the distribution of Pearson correlation coefficients from 10,000 repetitions of randomly splitting the filtered RT data of each individual subject into two halves. For each half, and each subject, the mean was computed. The vector formed by collecting means from the first half of all the subjects were correlated with the vector of means from the second half. The mean split-half correlation of Pro-point and Anti-point tasks were 0.985 ± 0.003 and 0.983 ± 0.003 SD, respectively.

To evaluate the reliability of RT measurements, a split-half reliability analysis was conducted. In this analysis, for each iteration, and each task, each subject’s RT data was split into two halves. The RTs included in each half were randomly selected. Then, for each subject, the mean RT for each half was computed. Thus, for each iteration, and each half, a vector of means from all the subjects was obtained. Pearson correlation coefficient is computed from the mean vectors of each half. The analyses utilized 10000 iterations. The results of these analyses are shown in Figure 3.

## Results

Figures 1A,B show the Pro-point and Anti-point response times that are binned according to the previously used ranges (Munoz et al., 1998). After model pruning, the only significant factors left in the mixed model analysis were Age (mean model: *F*(40,40) = 1.89, *p* = 0.02; median model: *F*(40,40) = 1.83, *p* = 0.03), Task (mean model: *F*(1,40) = 356.6, *p* < 0.001; median model: *F*(1,40) = 356.1, *p* < 0.001) and the interaction term of Age and Task (mean model: *F*(40,40) = 1.82, *p* < 0.03; median model: *F*(40,40) = 1.73, *p* = 0.04). Because there are no differences in results from mean and median models, we will use mean RTs for subsequent analyses. The effect of age on RT is greater on Anti-point (DRT = 126.7 ms and IRT = 247.3 ms) compared to Pro-point (DRT = 47.4 ms and IRT = 99.1 ms) task (note differences in vertical scale in Figures 1A,B). Note that DRT is defined over the early developmental age range whereas IRT is defined over the later aging range. The mean difference in DRT between Anti-point and Pro-point tasks is 79.3 ± 88.5CI (*t*(13) = 1.9, *p* = 0.07). The mean difference in IRT between Anti-point and Pro-point tasks is 148.2 ± 101.6CI (*t*(15) = 3.2, *p* = 0.007). It is also evident that RT varies in a quadratic manner with age for both the Pro-point and Anti-point tasks. The shortest mean RT are around the 15–22 age range (fitted curve minima at 21.5) for Pro-point and 23–29 age range (fitted curve minima at 27.8) for Anti-point tasks.

In the Pro-point task, the partial *F* analysis shown in Figure 2 (squares) indicates that the contribution of the quadratic regressor is significant (i.e., graph above zero) only up to an age of about 45 years. In our study, beyond this age, RT does not vary with age. Therefore, the full model (equation 1) fits shown in Figure 1A only utilized data up to the 40–49 bin. In contrast, in the Anti-Point task, the partial *F* analysis shown in Figure 2 (circles) indicates that the contribution of quadratic regressor is significant for almost all tested ages. The full model fit shown in Figure 1B thus utilized all the data.

The distribution of correlation coefficients in the split-half reliability analyses are shown in Figure 3. The mean correlation coefficients for Pro-point and Anti-point task are 0.985 ± 0.003 and 0.983 ± 0.003 SD, respectively.

## Discussion

The purpose of this study was to examine age-related changes on stimulus-driven and goal-directed functioning using novel tablet-based pointing tasks, modeled after Pro-saccade and Anti-saccade tasks utilizing eye-tracking technology. We found significant effects of age on both stimulus-driven and goal-directed functioning (*U* shaped curves). In addition, we report significant differences in the effect of age in the Pro-point and Anti-point tasks, indicating that advancing age differentially influences stimulus-driven and goal-directed processes. The measurements for both tasks are extremely reliable as indicated by exceptionally high correlations in the split-half reliability analyses and suggest that these tablet-based tasks may provide an inexpensive, quick, and more practical way of tracking sensorimotor and cognitive changes than other oculomotor methods.

### Non-linear Changes With Age

Analysis of the manual pointing responses shows that the effect of age on stimulus-driven and goal-directed functioning was nonlinear and *U*-shaped rather than linear, with the peak performance, identified by the quickest response times being present in late adolescence/early adulthood. This pattern is consistent with earlier pointing performance research done by Hertzum and Hornbæk (2010) which compared young, adult, and elderly subjects and showed that the adult subjects completed pointing trials quicker than the other two groups. Similarly, the results are also consistent with research measuring saccade dynamics across development which show peak performance in early adulthood (Munoz et al., 1998; Irving et al., 2006). Slower response times in the younger age groups are likely due to the time course of white matter brain development, which MRI studies have shown to increase at a steeper rate during adolescence (Paus, 2005; Mabbott et al., 2006; Lenroot et al., 2007). The progression of synaptic pruning and myelination in the PFC also becomes prominent during adolescence (Yakovlev and Lecours, 1967; Huttenlocher, 1979). Additionally, the slower response times in both the Pro-point and the Anti-point task for the older age groups is likely attributed to more widespread known neural changes during senescence and natural age related decline (Deary et al., 2009), including frontal, temporal, and parietal cortical regions (Lehmbeck et al., 2006).

### Changes in the Elderly: Goal-Directed vs. Stimulus-Driven Responses

Our results suggest that goal-directed functions continue to decline with age whereas stimulus-driven function decline appears to stabilizes around 50 years of age. However, the seeming stability of stimulus-driven performance in older adults may be explained by the relatively young age of the eldest cohort in our study (mean 61.3), which may not capture differences that occur later in life. Some previous work examining stimulus-driven function found no significant difference in the accuracy or duration of reaching movements to stationary targets between young and older adults (Sarlegna, 2006). However, other arm movement research suggests that stimulus-driven and goal-directed movements are susceptible to age dependent decline (Goggin and Meeuwsen, 1992; Rossit and Harvey, 2008; Hertzum and Hornbæk, 2010; Verneau et al., 2015), including work showing significant slowing in stimulus-driven movements, especially in healthy elder adults after the age of 70 years (Teeken et al., 1996). A similar heterogeneity of findings is found in the eye movement literature. That is, some studies on Pro-saccade eye movement latencies found no differences between young (mean 19–22 years) and older adult populations (mean 62–66 years). However, additional studies on Pro-saccade latencies in healthy, older populations show a significant slowing with age (Abel et al., 1983; Klein et al., 2000; Peltsch et al., 2011). Taking these findings in both arm and eye movements into account, it is likely that in older adults, especially beyond 70, we might also see some evidence of the slowing of stimulus-driven function. Nevertheless, our study importantly shows significant differences in the effect of age in stimulus-driven and goal-directed pointing tasks, indicating that advancing age differentially influences these processes. In addition, the RT changes with age were larger for Anti-point than Pro-Point. These findings imply that these processes (or some aspect of these processes) are subserved by different underlying mechanisms (Connolly et al., 2000; Day and Lyon, 2000). Prior pointing research examining learning in explicit (goal-directed) and implicit (stimulus-driven) pointing tasks also suggests that voluntary, goal-directed motor learning is slowed with age more than stimulus-driven learning (Verneau et al., 2015). These findings are also consistent with evidence in healthy aging suggesting that the largest changes in structure and function occur in the PFC (Cabeza, 2002; Nordahl et al., 2006). The dissociation between stimulus-driven and goal-directed performance with age provides insight into how these behaviors can help dissociate the neural changes that might occur in disease. Namely, Brooks et al. (2017) found that a specific pattern across stimulus-driven and goal-directed performance (i.e., increased antisaccade errors combined with slowed prosaccade latencies) appeared to be a useful marker for early differentiation among normal aging and various parkinsonian disorders.

### Possible Confounding Changes in the Elderly

It is possible that there are multiple underlying changes in the elderly. However, in order to be able to more carefully examine the trial to trial variability of pointing responses, an additional study with a larger number of trials would be required. Previous work in eye movements had reported that elderly individuals make significantly fewer or no express saccades (very fast stimulus-driven saccades; Munoz et al., 1998; Klein et al., 2000). Such a finding could also lead to slower or *increased* sensorimotor behavioral response times with age. However, more recent work suggests that the proportion of express saccades does not decrease with age (Peltsch et al., 2011). This study used the distribution of correct and error saccadic response times to define the express saccade epoch for each age group, which identified a broader distribution of these fast saccades (up to 200 ms). Although Peltsch et al. (2011) claimed that the proportion of express saccades did not correlate with increasing age group, their method for determining the *express epoch end* may have accidentally given the younger age groups, which had tighter (greater overlapping) distributions of express and regular latency saccades, an unfair advantage. That is, given that regular saccades have a broader variance and make up a larger proportion of all saccades, the greater the overlap of the two distributions (as occurs in the younger cohorts), a greater number of regular saccades will be included (counted) within the distribution of the express saccades, leading to the appearance of a constant proportion of express saccades with age. Hence, although they showed that express saccades do not decrease with age, it is possible that express saccades actually increase with age (see shaded regions in prosaccade column of Figures 2A,B vs. Figures 2D,E in Peltsch et al., 2011). However, given that Peltsch et al. (2011) also showed an increase in the latency of express and regular saccades with age, it is likely that even if there was an increased proportion of express saccades, it may not decrease mean Pro-saccade (or even mean express saccade) latencies. Hence, similar to saccades, it is possible that there may be an increased proportion of fast pointing movements (or increased response disinhibition), as well as an increased latency of pointing responses (or slowed stimulus-driven sensorimotor function) and that these multiple stimulus-driven aging factors could be canceling each other giving the appearance of stability. An additional study with a larger number of trials and more detailed analysis of the distributions would be able to determine if multiple, perhaps confounding, age-related changes in brain circuitry are occurring.

### Future Directions

Although the results from this study did show a strong relationship between age and goal-directed function, additional subjects in the younger age groups would be beneficial considering the variance in development at that point in life. Further, closer examination of older adults, both increasing the age range and greater number of trials, would help to determine if stimulus-driven manual performance truly remains stable across older age groups. In addition, given our age divisions in the present study, it is possible that larger groups and greater resolution of ages at the younger age groups would reveal small but significant gender differences consistent with previous work. Studies have shown that there are sex-related differences in brain volume with white matter volume increasing at a faster pace in males than females as a function of age (De Bellis, 2001; Lenroot et al., 2007). Nonetheless, our findings suggest close parallels with the eye movement literature and build upon the literature regarding pointing tasks, showing that age affects pointing task performance in a non-linear fashion, which is critical to helping establish the time course of stimulus-driven and goal-directed performance as measured with simple pointing responses.

## Conclusion

Current standards for establishing normative data concerning age related sensorimotor and cognitive functions involve expensive or difficult to use technology. However, the results from this study show that this new and simple tablet-based task is also effective in measuring and detecting age related differences in stimulus-driven and goal-directed functioning. Determining pathological development requires a firm understanding of the characteristics of the normal development of these brain functions. The results found in this study can provide a preliminary understanding of how these variables change with age and offer a standard for comparison of cognitive development in patients with abnormal neurophysiological development. Such simple tasks may be useful in differentiating parkinsonian disorders in the elderly (Brooks et al., 2017). The results in this study may also be helpful as an age-based preliminary understanding for comparison of cognitive development in adolescents with abnormal neurophysiological development, for example that may occur with repeated sub-concussive injury (Zhang et al., 2013; Koerte et al., 2017) or young adults with mild traumatic brain injury (Fischer et al., 2016). Equally important, perhaps especially in elderly individuals, these tasks can help identify intact functions, allowing individuals to capitalize on such abilities for the preservation of everyday competence and quality of life.

## Ethics Statement

Subjects provided written informed consent or assent with parental consent in accordance with the Declaration of Helsinki and were enrolled into a study approved by the Committee for the Protection of Human Subjects, the Institutional Review Board at the University of Texas Health Science Center at Houston.

## Author Contributions

EL collected the data, analyzed the data, and prepared and reviewed the manuscript. SL collected the data and prepared and reviewed the manuscript. SP analyzed the data and prepared and reviewed the manuscript. AS formulated the study, designed, collected the data and analyzed, and prepared and reviewed the manuscript. All authors contributed to draft parts of the work, have approved the final version, and agreed to be accountable for all aspects of the work.

## Conflict of Interest Statement

Co-authors AS and SP are named inventors of patents US-9,717,459, August 1, 2017 and US-9,949,693, April 24, 2018; and AS is member of CogNeuro Solutions LLC. The remaining authors declare that the research was conducted in the absence of any commercial or financial relationships that could be construed as a potential conflict of interest.
